# Telehealth-Based Music Therapy Versus Cognitive Behavioral Therapy for Anxiety in Cancer Survivors: Rationale and Protocol for a Comparative Effectiveness Trial

**DOI:** 10.2196/46281

**Published:** 2023-04-27

**Authors:** Kevin T Liou, Kelly M McConnell, M Beatriz Currier, Raymond E Baser, Jodi MacLeod, Desiree Walker, Camila Casaw, Greta Wong, Lauren Piulson, Karen Popkin, Ana Maria Lopez, Katherine Panageas, Joke Bradt, Jun J Mao

**Affiliations:** 1 Memorial Sloan Kettering Cancer Center New York, NY United States; 2 Miami Cancer Institute Miami, FL United States; 3 Society for Integrative Oncology Washington, DC United States; 4 Young Survival Coalition New York, NY United States; 5 Thomas Jefferson University Philadelphia, PA United States; 6 Drexel University Philadelphia, PA United States

**Keywords:** oncology, anxiety, cognitive behavioral therapy, music therapy, telehealth, cancer survivorship, mental health, digital health, mobile phone

## Abstract

**Background:**

Cancer survivors represent one of the fastest growing populations in the United States. Unfortunately, nearly 1 in 3 survivors experience anxiety symptoms as a long-term consequence of cancer and its treatment. Characterized by restlessness, muscle tension, and worry, anxiety worsens the quality of life; impairs daily functioning; and is associated with poor sleep, depressed mood, and fatigue. Although pharmacological treatment options are available, polypharmacy has become a growing concern for cancer survivors. Music therapy (MT) and cognitive behavioral therapy (CBT) are evidence-based, nonpharmacological treatments that have demonstrated effectiveness in treating anxiety symptoms in cancer populations and can be adapted for remote delivery to increase access to mental health treatments. However, the comparative effectiveness of these 2 interventions delivered via telehealth is unknown.

**Objective:**

The aims of the Music Therapy Versus Cognitive Behavioral Therapy for Cancer-related Anxiety (MELODY) study are to determine the comparative effectiveness of telehealth-based MT versus telehealth-based CBT for anxiety and comorbid symptoms in cancer survivors and to identify patient-level factors associated with greater anxiety symptom reduction for MT and CBT.

**Methods:**

The MELODY study is a 2-arm, parallel-group randomized clinical trial that aims to compare the effectiveness of MT versus CBT for anxiety and comorbid symptoms. The trial will enroll 300 English- or Spanish-speaking survivors of any cancer type or stage who have experienced anxiety symptoms for at least 1 month. Participants will receive 7 weekly sessions of MT or CBT delivered remotely via Zoom (Zoom Video Communications, Inc) over 7 weeks. Validated instruments to assess anxiety (primary outcome), comorbid symptoms (fatigue, depression, insomnia, pain, and cognitive dysfunction), and health-related quality of life will be administered at baseline and at weeks 4, 8 (end of treatment), 16, and 26. Semistructured interviews will be conducted at week 8 with a subsample of 60 participants (30 per treatment arm) to understand individual experiences with the treatment sessions and their impact.

**Results:**

The first study participant was enrolled in February 2022. As of January 2023, 151 participants have been enrolled. The trial is expected to be completed by September 2024.

**Conclusions:**

This study is the first and largest randomized clinical trial to compare the short- and long-term effectiveness of remotely delivered MT and CBT for anxiety in cancer survivors. Limitations include the lack of usual care or placebo control groups and the lack of formal diagnostic assessments for psychiatric disorders among trial participants. The study findings will help guide treatment decisions for 2 evidence-based, scalable, and accessible interventions to promote mental well-being during cancer survivorship.

**International Registered Report Identifier (IRRID):**

DERR1-10.2196/46281

## Introduction

### The Burden of Anxiety During Cancer Survivorship

With advances in oncology care, the number of cancer survivors in the United States is expected to increase dramatically and exceed 22 million by the end of this decade [1]. A meta-analysis identified anxiety as one of the most common mental health issues faced by cancer survivors [2]. Compared with the general population, cancer survivors experience higher rates of anxiety [2-5], and up to one-third of them experience clinically meaningful anxiety symptoms [3,5-9]. Characterized by restlessness, muscle tension, and worry that is difficult to control, anxiety is a highly disruptive symptom associated with poor sleep [10], depressed mood [11-13], and fatigue [14-17]. Anxiety also contributes to poor treatment adherence [18-20] and increased health care expenditures [21,22]. As a primary driver of poor quality of life among cancer survivors [23-26], anxiety is an important issue to address in this rapidly growing population.

Although pharmacological treatment options for anxiety are available [27], polypharmacy is a growing concern among cancer survivors [28-31]. Compared with the general population, cancer survivors have much higher rates of polypharmacy, with 64% taking ≥5 unique medications [30]. One-third of survivors receive at least 1 psychotropic medication (eg, anxiolytics and antidepressants), and nearly 1 in 6 survivors report using ≥2 classes of psychotropic medications [31]. Psychotropic polypharmacy is associated with poor quality of life, impaired physical and mental functioning, financial toxicity, and higher risk of side effects and drug interactions [31]. These challenges of polypharmacy highlight the need for nonpharmacological treatment options for anxiety during cancer survivorship.

### Cognitive Behavioral Therapy: An Effective First-Line Treatment for Anxiety

Cognitive behavioral therapy (CBT) is an evidence-based, nonpharmacological intervention delivered by licensed mental health professionals [32,33]. The American Society of Clinical Oncology (ASCO) [27] and the National Comprehensive Cancer Network (NCCN) [34] recommend CBT for treatment of anxiety in patients with cancer. Informed by the cognitive behavioral model of anxiety, CBT focuses on the relationship between thoughts, behaviors, and emotions and how thoughts and behaviors can exacerbate or reduce anxiety [35-37]. The therapeutic components of CBT consist of psychoeducation on anxiety, relaxation techniques, cognitive restructuring, and strategies for planning activity engagement and managing realistic worries [38,39]. Meta-analyses demonstrated moderate to large effects of CBT on anxiety symptoms in patients with cancer and cancer survivors relative to control conditions, with effect sizes ranging from 0.42 to 1.10 [40-43]. CBT is widely considered an effective first-line treatment for anxiety in people affected by cancer [27,34].

Despite the established effectiveness of CBT, studies have demonstrated that 20% to 25% of CBT participants fail to complete a full treatment course [44,45] and 37% do not achieve clinically meaningful improvements in anxiety symptoms [46,47]. Furthermore, social stigma surrounding psychotherapy exists in some demographic groups and cultures, and research shows that underserved racial or ethnic groups are more likely than White patients to delay, avoid, or drop out of mental health treatments [48,49]. These challenges of CBT highlight the importance of studying other nonpharmacological treatments that are not only effective but also culturally acceptable to diverse cancer survivors.

### Music Therapy: An Evidence-Based Anxiety Treatment With Increasing Availability at Cancer Centers

Music therapy (MT) is a nonpharmacological, evidence-based intervention in which board-certified music therapists engage patients in personally tailored experiences with music to achieve therapeutic goals [50]. These experiences range from music-guided relaxation to more active forms of musical engagement, including singing and improvising music [50]. The social-cognitive processing model of emotional adjustment in cancer is one of several possible models that informs the use of MT for anxiety management in cancer survivors [51]. Supported by empirical studies [52,53], this model conceptualizes the cancer treatment journey as a disruptive, trauma-like experience that must be cognitively processed in a supportive social context for healthy emotional adaptation to occur [51,52]. A growing body of research demonstrates the capacity of MT to influence these social-cognitive processes central to anxiety. Interactive music experiences have been shown to build social connections and promote a sense of belonging [54-56]. Prior studies also suggest that MT provides novel, creative outlets (eg, through songwriting) to cognitively process past traumatic experiences that may otherwise be difficult to verbalize [57,58]. In addition to its social-cognitive effects, musical engagement has been shown to modulate brain regions (ie, amygdala) [54] and biological systems (ie, hypothalamic-pituitary-adrenal axis [59-61] and autonomic nervous system [62-65]) responsible for emotional regulation and implicated in anxiety and mood disorders [66-69]. Importantly, ethnographic and phylogenetic research has identified music as a defining characteristic of humankind across many cultures worldwide [70,71]. The multicultural presence of music supports the potential of MT to resonate with a diverse population of cancer survivors.

MT has a growing evidence base for cancer symptom management [72-75] and is recommended by ASCO [76], NCCN [34], and the Society for Integrative Oncology [77,78] as a treatment option for anxiety in cancer populations. A recent Cochrane review (81 trials, N=5576) found that MT was associated with a large reduction in anxiety symptoms compared with usual care [79]; MT is also offered at approximately 75% of the National Cancer Institute (NCI)–designated Comprehensive Cancer Centers and 55% of community-based cancer programs [80]. Owing to its growing evidence base and availability, MT represents a promising alternative to CBT for the treatment of anxiety.

### Digital Transformation of Health Care During COVID-19: A Timely Opportunity to Improve Access to Mental Health Resources

In the past 2 decades, the percentage of US adults who use the internet has increased from 52% to 90% [81]. Approximately 59% to 79% have access to home broadband internet services [81] and 37% use smartphones as their primary access to the internet [82]. Cancer centers across the country are increasingly leveraging digital technology to monitor and manage symptoms remotely [83]. The COVID-19 pandemic accelerated these digital trends, and the use of videoconferencing for telehealth services increased by 8700% at the peak of the pandemic [84].

Although key disparities in digital access and literacy remain [85], the widespread adoption of videoconferencing offers a promising platform for reducing barriers to mental health services. Therapists have successfully used videoconferencing to deliver MT remotely to military populations [86-90] and patients with autism [91]. Therapists have also used videoconferencing to deliver MT services to inpatient [92,93] and oncology settings [94]. At the outset of the pandemic, the American Music Therapy Association and other MT professionals rapidly developed and implemented telehealth guidelines and resources to facilitate the telehealth-based delivery of MT services to patients isolated at home [92,95]. Many music therapists have shifted their clinical practices to web-based platforms [96,97]. There is also an extensive body of research (>100 trials) on internet-delivered CBT interventions [98,99], and studies have consistently demonstrated that CBT delivered remotely is as effective and acceptable as in-person treatments for anxiety [100]. Thus, both MT and CBT are equipped for the digital health care landscape, with unique potential for scalability to reduce barriers to mental health services.

### Gaps in the Evidence: Treatment Dilemmas Facing Patients and Clinicians in the New Digital Health Care Landscape

Although CBT is widely considered a first-line treatment for anxiety, not all individuals are able to complete a full treatment course [44,45] or achieve meaningful improvements in their symptoms [46,47]. People may also be reluctant to pursue CBT because of the sociocultural stigma surrounding psychotherapy in different groups and communities [48,49]. For individuals who do not respond or do not wish to pursue CBT, evidence is lacking on whether MT is an effective, noninferior treatment alternative to CBT.

To inform treatment decision-making, the American Psychological Association, in its resolution on the Recognition of Psychotherapy Effectiveness, has called for “continued and further research on the comparative effectiveness” of CBT and other psychotherapeutic interventions [101]. Furthermore, as health care continues to undergo rapid digital transformation, there is a critical need for additional research into telehealth interventions. Although CBT research has compared remote delivery versus traditional in-person formats, few studies have compared telehealth-based CBT with other telehealth interventions, such as MT [98,100]. In their systematic review of digital health interventions for cancer survivors, Harris et al [83] emphasized that “one size may not fit all” and there is a “need to identify who is most likely to benefit from digital interventions.” Comparative effectiveness research on different telehealth-based treatments (ie, CBT vs MT) is essential to guide timely and patient-centered decision-making and help patients and clinicians navigate this new digital health care landscape.

### The Music Therapy Versus Cognitive Behavioral Therapy for Cancer-Related Anxiety Trial: Study Aims and Hypotheses

The Music Therapy Versus Cognitive Behavioral Therapy for Cancer-related Anxiety (MELODY) trial seeks to address the following aims:

The primary aim is to determine the comparative effectiveness of telehealth-based MT versus telehealth-based CBT for anxiety and comorbid symptoms in cancer survivors.

- *Hypothesis 1(a)*: MT will be noninferior to CBT in treating anxiety symptoms among survivors at week 8 (end of treatment) and week 26 (long-term follow-up).
- *Hypothesis 1(b)*: Compared with CBT, MT will be associated with significantly greater improvement in fatigue co-occurring with anxiety.
- Exploratory hypothesis: Survivors may have unique experiences with MT and CBT for anxiety during cancer survivorship.

The secondary aim is to identify patient-level factors associated with greater anxiety symptom reduction after MT and CBT.

- *Hypothesis 2*: Specific sociodemographic characteristics (eg, age, sex, race, and education) or psychological attributes (ie, expectancy) will be associated with treatment response to MT or CBT.

## Methods

### Study Design

**Figure 1:**
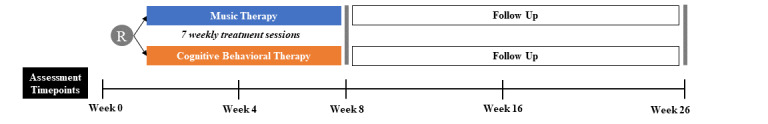
Study schema.

The MELODY study is a 2-arm, parallel-group, pragmatic randomized clinical trial that aims to compare the effectiveness of MT versus CBT for anxiety and comorbid symptoms in a diverse and heterogeneous sample of 300 cancer survivors (Figure 1). This study will be conducted at the Memorial Sloan Kettering Cancer Center (MSK), an NCI-designated Comprehensive Cancer Center, as well as the Miami Cancer Institute (MCI) and Drexel University. These sites were chosen in part for their potential to help recruit a diverse study population. Interventions will be delivered weekly via Zoom (Zoom Video Communications, Inc) over 7 weeks. Study assessments will be administered at baseline and at weeks 4, 8, 16, and 26. This study will be conducted in accordance with the Consolidated Standards of Reporting Trials (CONSORT) guidelines for nonpharmacological interventions [102], the reporting guidelines for music-based interventions [103], and the National Institutes of Health Protocol Template for Behavioral and Social Sciences Research [104]. This study has also been registered at ClinicalTrials.gov (NCT05215353).

### Ethics Approval

The study was approved by the institutional review board (IRB) at MSK on December 22, 2021 (IRB: 21-516). MSK serves as the IRB of record for both MCI and Drexel University.

### Eligibility Criteria

The eligibility criteria were designed to be broad and consistent with a pragmatic design while ensuring participant safety and scientific rigor. Participants are eligible if they are English- or Spanish-speaking, are aged ≥18 years, have a prior cancer diagnosis of any type or stage, are free of oncological disease, have a score of ≥8 on the anxiety subscale of the Hospital Anxiety and Depression Scale (HADS), report anxiety symptoms lasting at least 1 month, and have access to Zoom and a quiet or private location. Participants will be excluded if they had completed active cancer treatment (eg, surgery, radiation, and chemotherapy) less than 1 month before enrollment; however, participants receiving maintenance hormonal or targeted therapies will be allowed to enroll. Participants will also be excluded if they have active suicidal ideation, bipolar disorder, schizophrenia, or substance abuse; have a score of ≥10 indicative of cognitive impairment on the Blessed Orientation-Memory-Concentration; or received a treatment course of ≥7 MT or CBT sessions for anxiety symptoms within the last 6 months.

### Recruitment

This study will use a multimodal recruitment strategy that comprises population-based methods, clinician referrals, and community outreach and engagement, which are described as follows:

1. Population-based methods: MSK and MCI patient databases will be queried to identify survivors who meet basic eligibility criteria. MSK catchment areas include urban and suburban locations in New York, Long Island, and New Jersey, whereas MCI catchment areas include urban and suburban locations in South Florida with access to a large Hispanic population. Drexel University investigators will query the Pennsylvania Cancer Registry to identify potential participants from rural populations. A recruitment letter will be sent to the potential participants identified on these databases and registries. The recruitment letter will introduce the study and include instructions for interested persons to contact the study team.
2. Clinician referrals: potential participants will also be identified and referred by clinicians at MSK, MCI, or other health care settings. To facilitate referrals, the study team will educate clinicians about the study, focusing on those who care for cancer survivors.
3. Community outreach and engagement: information about the study will also be posted on social media outlets, ClinicalTrials.gov, and public-facing websites. The study team will also present the study at scientific conferences, cancer survivorship support groups, community organizations, and other patient advocacy groups.

The goal of this multimodal approach is to enroll a diverse sample that includes traditionally underrepresented sociodemographic groups. The recruitment strategies were developed jointly by the study team and the patient and stakeholder advisory board, which includes patient advocates, cancer survivors, community leaders, and clinical stakeholders. Diverse study representation will facilitate careful exploration of the differences between participant subgroups to inform personalized decision-making.

Once interested and potentially eligible participants are identified, the research staff will schedule them for an initial screening visit. If participants meet the basic eligibility criteria, they will then be scheduled for a telehealth visit with a study clinician to confirm their eligibility. If a participant is deemed eligible, the research staff will explain the study procedures and request informed consent. Once consent is obtained, the participants will complete the baseline assessments.

### Randomization

After completing the baseline assessments, participants will be randomized using MSK’s secure computer system that ensures full allocation concealment. Randomization will be 1:1 (MT:CBT) using randomly permuted blocks of random length, which are stratified by study site, current anxiety medication use (yes or no), and preferred language (English or Spanish). The principal investigator and biostatistician will be blinded to treatment assignments.

### Interventions

#### Treatment Course

Study participants will receive 7 weekly 60-minute MT or CBT sessions over the first 7 weeks. All sessions will be delivered remotely through Zoom’s Health Insurance Portability and Accountability Act (HIPAA)–compliant, encrypted, and passcode-protected videoconferencing software. Although the duration of these types of treatments may vary in real-world settings, this study will match the MT and CBT groups for patient-therapist contact time to ensure rigorous comparative effectiveness comparison.

#### MT Protocol

MT will be delivered by board-certified music therapists. The MT protocol is based on the neuroscience of music [54,105], the social-cognitive processing model [51,52], the scientific literature on MT [72], and the clinical and research experience of the study team [106]. Healthy psychological adjustment during cancer survivorship requires the processing of feelings and thoughts related to one’s cancer journey in a supportive context [51,52]. Therefore, the intervention protocol is structured to build patient-therapist rapport and allow for greater emotional expressivity and deeper cognitive processing. Guided by the social-cognitive processing model [51,52], the first few sessions will focus on building a trusting relationship with the therapist, whereas the later sessions will focus on the cognitive processing of cancer-related experiences and current fears, worries, and hopes, using playlist creation and songwriting as an outlet for reflection, expression, and meaning making. Research also suggests that patients prefer receptive modes of engagement during their initial exposure to MT [75,107]. As such, the early sessions will focus on receptive techniques (eg, music-guided stress management), and the later sessions will progress to more active MT techniques (eg, songwriting). The protocol includes homework activities in between sessions to promote self-management skills (eg, the use of music-guided deep breathing), strengthen social connections (eg, sharing meaningful songs with loved ones), provide outlets for cognitive processing (eg, writing or reflecting on themes for song lyrics), and serve as transitions to the subsequent sessions.

#### CBT Protocol

The CBT protocol is based on the scientific literature [32,33] and the clinical and research experience of the study team [108]. CBT therapists will include licensed social workers because they are the most commonly employed mental health providers in cancer care and, therefore, reflect the real-world practice and implementation of CBT in oncology settings [109]. Informed by the cognitive behavioral model of anxiety, CBT focuses on addressing the thoughts and behaviors that exacerbate or reduce anxiety [35-37]. The protocol consists of psychoeducation on anxiety, relaxation techniques, cognitive restructuring, and strategies for planning activity engagement and managing realistic worries [38,39]. These components target the somatic symptoms of anxiety (eg, muscle tension) as well as the thoughts (eg, “What if my next scan is bad?”) and behaviors (eg, excessive symptom monitoring) that trigger and exacerbate anxiety. Once identified, these problematic thoughts and behaviors are replaced by thoughts and behaviors that prevent and reduce anxiety. Each session will follow a consistent format that includes an overview of the session content, review of the homework exercise from the prior session, information on a new skill for managing anxiety, discussion of the upcoming homework exercise, and a plan for completing the exercise before the next session. Patients will receive a workbook with materials for each session and homework activities that promote practice of the skill learned during the session.

#### Interventionist Training and Treatment Fidelity

All interventionists will be trained about the specific research protocol and educated on the importance of adherence to the protocol methods. The interventionists will be either English speaking or bilingual in English and Spanish. The training of study interventionists will include didactic information on anxiety in cancer survivors and review of intervention materials. Interventionists will be provided with ongoing supervision over the course of the trial. During the trial, the CBT and MT sessions will be recorded and stored on secure, encrypted MSK servers. To ensure that study therapists adhere to the treatment protocol, document treatments appropriately, and maintain fidelity to the core functions of the intervention, the session recordings for the first 2 patients seen by a therapist will be reviewed. The review of session recordings will be based on treatment fidelity checklists outlining the core intervention components. If a therapist adheres to at least 80% of the treatment fidelity checklist items, then subsequent fidelity monitoring will be reduced to 2 randomly selected session recordings per patient. Deviations from the MT or CBT protocols will be discussed with study therapists during supervision meetings, and strategies will be suggested to minimize the number of deviations. Therapists who fail to adhere to at least 80% of the treatment fidelity checklist items will be retrained. Similar treatment fidelity strategies have been successful in other MT [106] and CBT trials [108,110] of patients with cancer conducted by the study team.

### Study Assessment Procedures

#### Overview

Patient-reported outcomes (Table 1) will be completed on the web using Research Electronic Data Capture (REDCap; Vanderbilt University) [111]. Participants will also have the option to complete these study assessments on paper or over the telephone with research staff. Study assessment materials will be available in English or Spanish. Patient-reported outcome measures for anxiety, comorbid symptoms, and quality of life have been validated in Spanish [112-117]. Where needed, Spanish translation of other assessments took place in standard forward and reverse translation.

**Table 1:** Summary of patient-reported outcomes.

Primary or secondary	Name of outcome	Validated instrument	Time points (weeks)
Primary	Anxiety	HADS^a^	0, 4, 8, 16, and 26
Secondary	Depression	HADS	0, 4, 8, 16, and 26
Secondary	Fatigue	BFI^b^	0, 4, 8, 16, and 26
Secondary	Insomnia	ISI^c^	0, 4, 8, 16, and 26
Secondary	Pain	BPI^d^	0, 4, 8, 16, and 26
Secondary	Cognitive dysfunction	FACT-Cog^e^	0, 4, 8, 16, and 26
Secondary	Quality of life	PROMIS–Global Health^f^	0, 4, 8, 16, and 26
Secondary	Treatment expectancy	METE^g^	0 and 8
Secondary	Treatment preference	Treatment preference Scale	0 and 8
Secondary	Music reward	BMRQ^h^	0
Secondary	Mental health stigma	SSRPH^i^	0
Secondary	Generalized anxiety disorder	GAD-7^j^	0

^a^HADS: Hospital Anxiety and Depression Scale.

^b^BFI: Brief Fatigue Inventory.

^c^ISI: Insomnia Severity Index.

^d^BPI: Brief Pain Inventory.

^e^FACT-Cog: Functional Assessment of Cancer Therapy–Cognitive Function.

^f^PROMIS–Global Health: Patient-Reported Outcomes Measurement Information System–Global Health.

^g^METE: Mao Expectancy of Treatment Effects.

^h^BMRQ: Barcelona Music Reward Questionnaire.

^i^SSRPH: Stigma Scale for Receiving Psychological Help.

^j^GAD-7: Generalized Anxiety Disorder 7-Item Scale.

#### Anxiety

The primary outcome is severity of anxiety symptoms, as assessed using the HADS anxiety subscale. The reliability, validity, and factor structure of this 7-item subscale has been established in patients with cancer with a Cronbach α of .83 [118,119]. A score of ≥8 indicates the presence of anxiety symptoms. Research has identified a minimal clinically important difference (MCID) of 1.7 points [120]. The HADS anxiety subscale will be administered at baseline and at weeks 4, 8, 16, and 26.

#### Comorbid Symptoms and Quality of Life

Given that anxiety is strongly associated with depressed mood [11-13], fatigue [14-17], insomnia [10], pain [121], and cognitive dysfunction [122], these comorbid symptoms will be assessed using validated instruments. Depressive symptoms will be assessed using the HADS depression subscale (Cronbach α=.79) [118,119]. Fatigue will be assessed using the Brief Fatigue Inventory (BFI), a 9-item scale that has been validated in cancer populations with a Cronbach α of .96 [123]. Insomnia symptoms will be assessed using the Insomnia Severity Index [124], a 7-item instrument validated in cancer populations with a Cronbach α of .90 [125]. Pain severity and pain-related interference will be assessed using the Brief Pain Inventory (BPI). The BPI has been demonstrated to be a reliable, valid, and responsive measure with a Cronbach α of .77 to .91 [126]. Cognitive difficulties will be assessed using the Functional Assessment of Cancer Therapy–Cognitive Function (FACT-Cog), version 3, a 37-item questionnaire with 4 subscales (perceived cognitive impairments, impact on quality of life, comments from others, and perceived cognitive abilities). The FACT-Cog is a reliable instrument validated in cancer populations with a Cronbach α of .89 [127]. The FACT-Cog instrument will only be administered to participants who reply “YES” to the following question at baseline: “Are you experiencing difficulties with memory, concentration, or other cognitive abilities?” As anxiety is a key determinant of quality of life in cancer survivors [23-26], the Patient-Reported Outcomes Measurement Information System–Global Health (PROMIS–Global Health) will also be administered. This validated measure contains 2 domains related to quality of life: mental health (Cronbach α=.86) and physical health (Cronbach α=.81) [128]. These measures will be administered at baseline and at weeks 4, 8, 16, and 26.

#### Treatment Expectancy

The Mao Expectancy of Treatment Effects (METE) is a 4-item instrument originally developed as the Acupuncture Expectancy Scale to measure outcome expectancy. It has demonstrated validity and reliability (Cronbach α=.82) and is correlated with patient self-reported efficacy and satisfaction [129-131]. Prior research has also demonstrated that outcome expectancy is associated with treatment response [129,132,133] and can, therefore, help inform personalized treatment decisions. The score ranges from 4 to 20, with higher scores indicating greater expectancy of benefit. This measure has been adapted for the MELODY study to assess patient expectancy of MT and CBT. The METE will be administered at baseline before receiving treatment. It will also be administered at week 8 to explore whether the expectancy changes after the treatment process.

#### Treatment Preference

As participants may have different preferences toward mental health treatment based on health beliefs or prior experiences with interventions, they will be asked at baseline whether they prefer MT or CBT or none [134]. Their preferences will also be assessed at week 8 to explore whether preferences change after their experiences with either MT or CBT.

#### Music Reward

Prior research has demonstrated variability in how people derive reward and pleasure from music, which may affect their experience with MT [135]. Therefore, the Barcelona Music Reward Questionnaire will be administered at baseline to assess the level of reward associated with music. This instrument has been validated with a Cronbach α of .92 [135].

#### Stigma of Receiving Psychological Help

In some groups and cultures, there is stigma associated with mental health needs, which may affect treatment experiences [48,49]. Therefore, the Stigma Scale for Receiving Psychological Help will be administered at baseline. This instrument has been validated (Cronbach α=.72) in different cultures to assess the stigma associated with receiving psychological health from mental health providers [136,137].

#### Covariates

Sociodemographics (eg, age, sex, race or ethnicity, and education) and other relevant clinical characteristics (eg, cancer type, stage, treatment history, and time since cancer diagnosis) will be collected at baseline. The Generalized Anxiety Disorder 7-Item Scale (GAD-7) will also be administered as a diagnostic screen for generalized anxiety disorder at baseline [138]. The use of medications prescribed for anxiety (eg, antidepressants, anxiolytics, hypnotics, and sedatives) will be tracked using weekly medication diaries at weeks 0, 8, and 26.

#### Semistructured Interviews

It may be difficult to capture the impact of MT and CBT using quantitative measures alone. Methodological experts recommend mixed methods approaches to understand patient experiences with health interventions [139-141]. Thus, qualitative method specialists will conduct 45-minute, semistructured individual interviews over the phone at week 8 (end of treatment). To reach thematic saturation from interviews, the research team will purposively sample 30 survivors in each treatment arm to be interviewed (N=60), aiming for balance across age, sex, race, ethnicity, and treatment response. Interviews will be conducted shortly after the intervention period to enhance the recall of personal experiences during therapy sessions. Interviews will be conducted using a semistructured guide that covers the following topics: (1) acceptability: treatment satisfaction, usefulness of the intervention, barriers and facilitators to session attendance, and adherence to and engagement with between-session assignments; (2) impact on anxiety symptoms and coping: perceived benefits and harms of the intervention on anxiety and comorbid symptoms, how one copes with stress and anxiety, integration of learned skills into daily life, and plans for maintaining anxiety management skills; (3) digital literacy and experience: views toward technology, comfort level with telehealth, and transfer of acquired digital skills to other aspects of daily living; and (4) unexpected experiences: unanticipated benefits, harms, or insights from engaging with the interventions. The interview guide will include flexibility in asking probing questions to enable participants to elaborate on their experiences. All the interviews will be audio-recorded and transcribed for thematic analysis.

### Analytical Approach

#### General Description

Analyses will be conducted using the intention-to-treat principle (ie, participants will be analyzed according to their randomly assigned treatment group regardless of dropout or treatment adherence status). Given that CBT is widely recognized as a first-line therapy for anxiety, the study team opted to test the noninferiority of MT to CBT, with the goal of understanding whether MT can be an appropriate treatment option for patients who lack CBT access or do not wish to pursue CBT. To provide complementary information for treatment decision-making, the study team will also test which intervention is superior for addressing fatigue and other symptoms co-occurring with anxiety. For all specific aims, the main analytical tool will be a linear mixed-effects model (LMM) because the primary outcome (anxiety) and secondary outcomes (fatigue, depression, insomnia, pain, cognitive dysfunction, and health-related quality of life) are repeated continuous outcomes over time [142]. This statistical procedure takes into account within-subject correlations from repeated measurements in the same subjects and allows the estimation of between-group differences without necessitating the exclusion of participants with missing data. The general template of each LMM will model the outcome as a function of the treatment arm and assessment time (categorical), controlling for the randomization stratification variables (baseline anxiety medication use, preferred language, and study site) and including a subject-specific random intercept. The general LMM template will be tailored to test the specific aim hypotheses by adding interaction terms (eg, time-by-intervention) and additional covariates of interest to the model and by reparametrizing the assessment time variable to focus on specific contrasts.

#### Aim 1—Hypotheses 1(a) and 1(b)

Outcome measure trajectories will be plotted by randomization arm over time. Each outcome measure will be summarized at each assessment time by treatment arm using descriptive statistics. Comparisons between randomization arms with respect to changes in symptom outcomes will be based on specific coefficients from time-by-arm interactions added to the general LMM template described above in the *General Description* section. Specifically, the model will include all time points (categorical) at which the outcome was assessed (baseline and weeks 4, 8, 16, and 26), and the time-by-arm interaction will include multiple coefficients corresponding to the time-by-arm interaction at each discrete postbaseline time point. However, statistical inferences will focus on the interaction coefficients at 8 and 26 weeks, which are interpreted as the differences between arms in change from baseline to 8 and 26 weeks, respectively. For hypothesis 1(a), the 2 primary end points are HADS anxiety score changes at 8 weeks and 26 weeks. On the basis of the LMM coefficients for the time-by-arm interaction at weeks 8 and 26, 2 tests will be performed to determine whether MT is noninferior to CBT at reducing the HADS anxiety subscale score within a noninferiority margin of 0.35 SD for both comparisons. The noninferiority of MT to CBT will be tested at a significance threshold of *P*<.025 for each comparison, controlling the overall type I error at *P*=.05 for the primary end point comparisons. For hypothesis 1(b), a similar LMM evaluating BFI scores over time will be used to test whether the MT arm had significantly greater improvement in fatigue (BFI score) compared with the CBT arm at weeks 8 and 26. In contrast to the primary end point noninferiority comparisons, these tests will be superiority tests. For other comorbid symptoms (eg, depression and insomnia) and quality of life, the methods described for hypothesis 1(b) will be used to compare the treatment arms. Given that a small number of cancer survivors may experience disease recurrence during the 26-week study, sensitivity analyses will be performed, excluding those individuals who experienced a recurrence during the study period. These sensitivity analyses will not replace the primary analysis.

#### Aim 2—Heterogeneity of Treatment Effect

An essential part of patient-centered care is recognizing that not all patients will respond to treatments the same way. As such, exploratory, hypothesis-generating heterogeneity of treatment effect (HTE) analyses will be conducted to identify patient-level factors associated with treatment response to either MT or CBT by incorporating 6 relevant variables (sex, race, ethnicity, education, outcome expectancy, and time since cancer diagnosis) and variable-by-intervention interaction terms in the mixed-effects model described above in the *Aim 1—Hypotheses 1(a) and 1(b)* section. These 5 exploratory LMM-based analyses will be guarded against inflated type I error owing to multiple testing by adjusting the variable-by-intervention interaction *P* values for the false discovery rate [143,144]. This type of subgroup analysis, although patient centered, may need to be interpreted with caution and cannot replace the primary analyses. The evaluation and reporting of HTE analyses will be based on the approach proposed by Kent et al [145]. However, recognizing other factors may also contribute to treatment responses, the study team will also apply Bayesian [146,147] and machine learning [148,149] methods, which can identify HTE and subgroups based on multiple variables simultaneously and are potentially more powerful than traditional univariate methods.

#### Qualitative Analyses

All interviews will be transcribed verbatim and imported into MAXQDA 2022 (VERBI Software) for analysis. The semistructured interviews will be analyzed using theoretical thematic analysis procedures as per the guidelines by Braun and Clarke [150,151]. Such an analysis is aimed at identifying patterns driven by an a priori theoretical framework or research questions. Themes will be identified using a semantic approach in which themes are derived from “the explicit meaning of the data and the analyst is not looking for anything beyond what a participant has said” [150]. Two coders will code the interview transcripts. To ensure the trustworthiness of the analysis, the primary coder will code each transcript, whereas the secondary coder will code 30% of the transcripts. Coding discrepancies will be resolved through discussion until consensus is reached. The codes will then be organized into categories, reviewed, and subsequentially organized into broader themes. Interviews from each treatment arm will be analyzed separately and then compared for convergence and divergence. The themes will be linked to patient-reported outcomes to explore whether specific themes vary according to the magnitude of change in the HADS anxiety score. To augment the HTE analyses, the study team will also explore whether qualitative themes differed by subgroup (eg, sex, race, ethnicity, education, outcome expectancy, and time since cancer diagnosis) within each treatment group.

#### Missing Data

As the only certain way to avoid biases from missing data is to collect complete data [152], the study team will strive to minimize missing observations by using study procedures that reduce participant burden. Participants who experience difficulties completing assessments on the web will have the option to complete assessments on paper or over the telephone with the research staff. Participants with time constraints or other barriers to completing outcome assessments will be given the option to complete only the primary outcome, that is, the HADS scale. Participants who withdraw from treatments will be encouraged to continue completing the outcome assessments. Reasons for withdrawal will be recorded for those who completely withdraw from the study. Given that missing data are inevitable in a prospective study, sensitivity analyses will be performed to assess the impact on the results of adjusting for patient disease progression or death, and analytical strategies that are as robust as possible to data losses will be applied. The study team will first explore whether missingness is associated with observed variables (eg, randomization arm and baseline outcome measures) by comparing patients with complete and incomplete data. Notably, the LMMs described previously will include patients with incomplete data under the missing at random assumption. However, the exploration of the data may deem the missing at random assumption to be inappropriate. Multiple imputation and pattern mixture models are well-established methods for addressing these issues [153,154]. Sensitivity analyses will be performed to evaluate the robustness of the LMM results by refitting the models after imputing the missing week 8 and week 26 outcomes using multiple imputation.

#### Sample Size and Power

The sample size is based on the primary hypothesis that MT is noninferior to CBT for anxiety reduction at weeks 8 and 26. Two tests will be performed to determine whether MT is noninferior to CBT at reducing HADS anxiety at weeks 8 and 26, each with a noninferiority margin of Cohen *d*=0.35 SD for both comparisons. These comparisons will be based on the LMM coefficients for the time-by-arm interaction at weeks 8 and 26; however, the power calculation is based on 2-sample *t* tests of differences between the arms in their change scores. Therefore, the power estimate is conservative, that is, in the LMM-based analysis, there will be slightly higher power than the power calculation presented below, with all other assumptions held constant. Assuming 150 participants are randomized to each arm, 15% attrition, and a 1-sided significance threshold of *P*=.025 (controlling the overall type I error at 0.05 for our 2 primary end point comparisons), there will be 80% power to find MT noninferior to CBT with respect to the HADS anxiety subscale scores at weeks 8 and 26 within a noninferiority margin of Cohen *d*=0.35 SD.

The noninferiority margin was informed by a large study that used multiple rigorous methods to empirically estimate a difference of 1.7 points as the MCID for the HADS scales [120]. In preliminary data from CBT recipients with baseline HADS anxiety subscale scores ≥8, the SD for the HADS anxiety subscale score was 4.2 at week 8 [110]. In addition, in a large acupuncture trial of cancer survivors, among 159 patients with baseline HADS anxiety subscale scores ≥8, the HADS anxiety subscale score SD was 4.0 during follow-up [155,156]. On the basis of these studies, it is assumed that the HADS anxiety subscale score SD will be approximately 4.2. Dividing the raw-score MCID of 1.7 points by 4.2 yields a standardized difference, that is, Cohen *d*=0.40. The noninferiority margin of Cohen *d*=0.35 SD is smaller than the standardized MCID (Cohen *d*=0.40) of the HADS anxiety subscale; as such, the noninferiority margin and statistical approach will find MT to be noninferior to CBT only if MT is not meaningfully worse than CBT.

Regarding the sample size for the qualitative analyses, the number of participants required to draw meaningful conclusions from semistructured interviews is determined by saturation, that is, the point at which existing themes are fully understood and no new themes emerge through further data collection [157]. Prior research has indicated that thematic saturation can typically be obtained with <15 interview participants, although these findings were based on relatively homogeneous samples [158]. Given that this study is enrolling a more heterogeneous population, the plan is to conduct 30 interviews in each treatment arm to better capture the diverse perspectives.

## Results

The MELODY study was funded by the Patient-Centered Outcomes Research Institute in July 2021. The IRB at MSK approved the study protocol in December 2021. The first study participant was enrolled in February 2022. As of January 2023, a total of 151 participants have been enrolled. Enrollment is expected to be completed by February 2024, and data collection is expected to be completed by September 2024.

## Discussion

Cancer survivors represent one of the fastest growing populations in the United States [1]. Unfortunately, nearly 1 in 3 survivors experience anxiety symptoms as a long-term consequence of cancer and its treatment [3,5-9]. Although pharmacological treatment options are available, polypharmacy has become a growing concern for cancer survivors [28-31]. CBT is an established first-line treatment for anxiety [32,33,159], but not all individuals respond or wish to use CBT [45,47-49]. Although MT has a growing evidence base for anxiety in cancer populations [72-75], it remains unclear how MT compares with CBT as a treatment alternative. The MELODY study will address this evidence gap by determining the comparative effectiveness of telehealth-based MT and telehealth-based CBT for anxiety in cancer survivors. This study will intentionally support enrollment access for racially and ethnically diverse populations of English- or Spanish-speaking survivors from urban, suburban, and rural settings to improve the generalizability of the findings and to inform future efforts to reduce the disparities in survivorship care. By focusing on telehealth-based interventions, this study will capitalize on the accelerating digital trends in health care and society at large, helping patients and clinicians to navigate a growing array of remotely delivered care options.

Despite its novelty and strengths, the MELODY study has some limitations. First, the study lacks a usual care or waitlist control group and therefore cannot control for Hawthorne effects or regression to the mean; however, both MT [72-75] and CBT [40,43,160] have demonstrated effectiveness for anxiety symptoms in cancer populations. Second, although the study participants are screened for the presence of generalized anxiety disorder, they do not undergo a formal diagnostic interview for psychiatric disorders. Third, this study focuses on treating anxiety symptoms rather than an anxiety disorder. However, cancer care guidelines are often based on symptom severity rather than psychiatric diagnoses [161]. Furthermore, research indicates that anxiety symptoms are associated with poor quality of life and other problematic outcomes in cancer populations, even if symptoms do not meet the criteria for an anxiety disorder [23,162]. Thus, by focusing on symptoms, this study will align closely with clinical guidelines and enroll more patients who could potentially benefit from interventions.

In conclusion, the MELODY study is the first and largest randomized clinical trial to compare the short- and long-term effectiveness of remotely delivered MT and CBT for anxiety symptoms in cancer survivors. This trial has the unique potential to produce timely findings on 2 evidence-based, scalable, and accessible interventions to promote mental well-being during cancer survivorship.

## Appendix

Multimedia Appendix 1 Peer-review report from PCORI Funding Announcement: Assessment of Prevention, Diagnosis, and Treatment Options - Comparative Effectiveness Research (Patient-Centered Outcomes Research Institute, USA).

## Abbreviations

ASCO: American Society of Clinical Oncology
BFI: Brief Fatigue Inventory
BPI: Brief Pain Inventory
CBT: cognitive behavioral therapy
CONSORT: Consolidated Standards of Reporting Trials
FACT-Cog: Functional Assessment of Cancer Therapy–Cognitive Function
GAD-7: Generalized Anxiety Disorder 7-Item Scale
HADS: Hospital Anxiety and Depression Scale
HIPAA: Health Insurance Portability and Accountability Act
HTE: heterogeneity of treatment effect
IRB: institutional review board
LMM: linear mixed-effects model
MCI: Miami Cancer Institute
MCID: minimal clinically important difference
MELODY: Music Therapy Versus Cognitive Behavioral Therapy for Cancer-related Anxiety
METE: Mao Expectancy of Treatment Effects
MSK: Memorial Sloan Kettering Cancer Center
MT: music therapy
NCCN: National Comprehensive Cancer Network
NCI: National Cancer Institute
PROMIS–Global Health: Patient-Reported Outcomes Measurement Information System–Global Health
REDCap: Research Electronic Data Capture
