# Supplementary material for: Telehealth-Based Music Therapy Versus Cognitive Behavioral Therapy for Anxiety in Cancer Survivors: Rationale and Protocol for a Comparative Effectiveness Trial
Source: JMIR Res Protoc. 2023 Apr 27;12:e46281. doi: 10.2196/46281 (PMC10176150; doi:10.2196/46281)
Supplement: Multimedia Appendix 1 [file resprot_v12i1e46281_app1.pdf]

PATIENT-CENTERED OUTCOMES RESEARCH INSTITUTE
SUMMARY STATEMENT
(Privileged Communication)

**Principal Investigator:**	Jun Mao
**Organization:**	Memorial Sloan Kettering
**Project Title:**	Music Therapy vs. Cognitive-Behavioral Therapy for Cancer-related Anxiety (MELODY)
**PCORI Funding Announcement:**	Assessment of Prevention, Diagnosis, and Treatment Options
**Review Cycle:**	Cycle 3 2020
**Request ID:**	21044

NOTE: PCORI's Merit Review process includes written online critique and in-person discussion phases. All applications go through the online written critique phase, but only a subset continue to the in-person discussion phase.

If an application does not progress to in-person discussion, the Summary Statement includes only the written online critiques.

If an application progresses to in-person discussion, the Summary Statement includes in-person panel discussion notes, final average overall score, and written online critiques.

AVERAGE OVERALL SCORE: 29

In-Person Review Discussion Notes:

Strengths:

- This randomized controlled trial aims to determine the comparative effectiveness of virtual music therapy versus virtual cognitive-behavioral therapy (CBT) for anxiety and co-morbid symptoms in cancer survivors. It proposes a robust study design to answer a question of importance to patients.
- There is a strong conceptual framework.
- Both interventions are evidence-based and backed by guidelines. Reviewers perceived the proposed virtual delivery of the interventions as practical and appropriate especially given the context of the current pandemic. These are major strengths.
- Recruitment sites are culturally diverse, which will assist with generalizability. This is a major strength.
- The investigative team is strong and has experience with studies of this scope, size, and complexity. Their track record of successfully completing similar studies with low attrition, limited missing data, and high rates of participation among underrepresented groups was perceived as a major strength by reviewers.
- Strong patient-centeredness is evident in the responsiveness to patient input through the choice of comparators (exploring a non-CBT option), focusing on anxiety symptoms rather than diagnosis, and in the plans to use the

Spanish version of the Patient-Reported Outcomes (PRO) instruments.

Weaknesses:

- The reviewers identified minor to moderate weaknesses in the study design.
    - The definition of "cancer survivor" lacks detail (e.g., is it defined by remission or by cancer-free period). This can also vary across the different types of cancers being assessed. This is a minor, fixable weakness.
    - The definition of what constitutes music therapy needs additional clarification (e.g., that the intervention is appropriate for non-musicians, that clarity around where/whether music therapy overlaps with CBT, and that the choice of music will be tailored culturally). This is a minor, fixable weakness.
- The reviewers identified minor weaknesses in the analytic plan:
    - The proposed instrument for the assessment of assumption of normality may be inappropriate and skew results. This can impact how easily the mixed-effects model can be applied.
    - Sample size calculations are unclear.
    - It is unclear why the study proposes stratifying by language. Stratification by site was discussed as an alternative.
- The focus on Memorial Sloan Kettering and its affiliate site in Miami may limit generalizability and scalability in lower-resourced institutions/settings. This is a minor weakness.
- Effort designated for the senior statistician is low, and the expertise of the lead music and CBT therapist could not be evaluated because specific individuals to fill these roles have not been named yet. These are minor, fixable weaknesses.
- Access to the virtual interventions may be problematic for persons in low-resourced communities (e.g., those not having Internet or a device), and the financial incentive for participation is low. These are minor weaknesses.
- The study lacked caregiver or family stakeholder involvement and the number of stakeholder engagement meetings is low. The two patient partners are paid different rates for the same expertise. These are minor weaknesses.

Additional Comments:

It is unclear why 7 sessions/doses were chosen given the background literature cited, which used 14 sessions. There was significant discussion of the appropriateness of the 7 session dose. The rationale was not justified, and reviewers were inconclusive on whether this was adequate.

NOTE: The above In-Person Review Discussion Notes are a summary of the in-person reviewer group discussion.

Online Reviewer Critiques

The below reviewer critiques were written by individual panel members assigned to review this application prior to the in-person discussion and were not altered post discussion. These reviewer critiques might not necessarily reflect the position of the reviewers at the close of the group discussion.

Reviewer 1:

Criterion 1: Potential for the study to fill critical gaps in evidence (Scientist Reviewers)

Strengths:

- The proposal indicates that anxiety is commonly experienced by many cancer survivors (major).
- The proposal indicates that cognitive behavioral therapy (CBT) can be effective for anxiety but there can be stigma around CBT. The proposal notes how CBT requires mental and emotional stamina and can therefore be too demanding. CBT can be effective but not for all people and the application proposes that an engaging comparison condition is warranted (major).
- The proposal highlights that there is general evidence for music therapy (MT) in the treatment of anxiety and results of a Cochrane review (conducted by one of the authors of the proposal) support music therapy oncology populations. However, there is a lack of MT research for anxiety in cancer survivors, a growing group of people due to improvements in technology and treatment. While the proposal notes that CBT has strong empirical support, many people may not want CBT as it can be demanding, stigmatizing, and requires mental and emotional stamina. MT may require less of these factors and cancer survivors may be more likely to request, accept, and be actively involved in MT (as indicated in the MT literature and noted in the proposal). This application seeks to address the treatment of psychosocial factors cancer survivors experience using music therapy in a virtual delivery format therefore enhancing accessibility to underserved communities (major).
- Based on the cited Cochrane Review of MT for oncology and other related research, the proposal identifies that there is a critical gap in the literature about virtual MT for anxiety with cancer survivors and that virtual MT may be an optimal intervention to compare with virtual CBT. The results of the proposed study would fill that critical gap in the literature (major).

Weaknesses:

- None in this section.

Criterion 2: Potential for the study findings to be adopted into clinical practice and improve delivery of care (All Reviewers)

Strengths:

- The application noted that music therapists, cancer centers, and cancer survivors would be able to use study findings and advocate for virtual MT as a treatment (major).
- The proposal notes how CBT requires mental and emotional stamina and can therefore be too demanding. As such, the application provides information on the need for this study from end-users.
- The proposal's research findings have the potential to provide valuable data that would be used to inform key

stakeholders' decision making. Administrators would hire music therapists and end-users would request services. The treatment design is based on evidence and, if positive results occur, other practitioners could reproduce those positive results (moderate).

- The application notes a comprehensive plan to disseminate findings through local and national media, social media, newsletters, and professional conferences. Dr. Bradt will present results at the American Music Therapy Association conference. The dissemination plan will reach patients and stakeholders through the About Herbs newsletter (over 10,000 subscribers and millions of website visits each year) (moderate).

Weaknesses:

- MT is sometimes considered a "best buy" – music therapists, while highly trained, are typically less expensive than other psychosocial interventionists. As such, MT could have the potential to lead to improvements in cancer survivors across the country and result in greater access to effective treatment for cancer survivors. These were not made explicit in the application but constitute fixable items in the application (minor).
- A potential barrier to this study is that there are only about 9000 Board-Certified Music Therapists in the United States; this could limit access to effective treatment should the virtual MT intervention be non-inferior to virtual CBT (minor).
- The study will take place virtually but is based in New York, New Jersey, and Miami; While New York, New Jersey, and Miami have diverse populations, the proposal does not provide opportunities for other parts of the United States (i.e., Midwest, west coast) to be represented? (minor)
- The application did not explicitly mention publishing results in specific refereed journals (i.e., *Cancer* or another related high impact oncology journal) but this is inferred due to the academic publishing track records of the authors. While the application did not mention specific academic journals, the authors are experienced scholars and have the expertise to select the best journals for the most impact (minor).

Criterion 3: Scientific merit (research design, analysis, and outcomes) (Scientist Reviewers)

Strengths:

- CBT has strong theoretical and empirical support as noted in the application. The virtual MT in the study will be informed by the social cognitive processing model of emotional adjustment of cancer. The variables are relevant and germane to cancer survivorship (major).
- The proposal is a 2 arm randomized comparative effectiveness study of virtual MT and virtual CBT on anxiety. Other related secondary variables include depression, fatigue, insomnia, and quality of life. These are all relevant constructs for cancer survivors. The use of virtual delivery through HIPPA compliant Zoom may increase access to services (major).
- The patient population (adult cancer survivors) and setting (virtual delivery) is appropriate for this study. The virtual delivery format is especially relevant given COVID and participants who may be immunocompromised (major).
- Randomization will be 1:1 (CBT:MT) in permuted blocks of random length and stratified by anxiety medication (yes or no) and language (English or Spanish) (major).
- CBT and MT have standardized semi-fixed protocols with definitions (major).
- The application specifies rigorous methods, including masking when possible, that are congruent with PCORI Methodology Standards. The overall study design – 2 arm comparative effectiveness study with two follow-up measures to determine maintenance of treatment gains – is justified given the existing literature and gap in the literature (moderate).
- MT and CBT were adequately described. Standardized semi-fixed protocols were included in the appendix with definitions (major).
- The measures of anxiety, depression, fatigue, insomnia, and mental and physical health are relevant to the study population. Cronbach's alpha values were provided for each measure and these values were strong. Most of the

measures are relatively brief, indicating that the application recognizes fatigue may be a factor given the population. Participants are more likely to complete brief instruments and this aspect of the study may lessen attrition (moderate).
- There is also a qualitative component to better understand the service users' experiences in CBT and MT. Theoretical thematic analysis will be used to analyze these data (major).
- Power analyses were conducted, sample sizes are appropriate, and, given the experience and expertise of the proposal authors and the clinical sites, the project is feasible. Providing financial incentive to participants will help attrition. All plans are realistic (major).
- Thorough application and literature review and supportive scholarship; There were 170 different references (minor).

Weaknesses:

- The application did not specifically state if MT and CBT were to be delivered in group or individual formats. It is inferred from the application and protocols (appendix) that treatments will be delivered in individual formats. Group MT and group CBT could potentially allow for more normalization, universalization, and social connectedness between participants who are cancer survivors. There are major logistical hurdles in group formats and it is more difficult to control the treatment, so this is not a major concern in the application. Moreover, individual therapy also allows the therapist to better tailor the treatment to the service user and removes many confounding variables associated with group dynamics (minor).
- While not a health-related outcome, perhaps including therapeutic alliance as an outcome measure might provide an interesting comparison between MT and CBT. Although not part of the research question, perhaps including alliance as a measure would distinguish the treatments. As there are barriers associated with CBT, perhaps including an alliance measure might quantitatively distinguish MT and CBT conditions. In the literature, alliance is often a predictor of therapeutic outcome. Relational factors may emerge in the qualitative/interpretivist arm of the study (minor).
- The dose of each CBT and MT is 7 sessions over 7 weeks. There was no information in the application on why 7 doses were chosen. Usual evidence-based treatment courses of CBT for major depressive disorder are 14 weeks. While the intervention protocols are theoretically-informed, the dose may not be enough to induce positive – and enduring – change for participants. There was no supporting literature in the proposal that supported the rationale for 7 dose treatment. Perhaps this omission was an oversight and constitutes a fixable item in the application (minor).
- The virtual delivery format limits the MT interventions (i.e., improvisation and instrument playing do not typically work well in virtual delivery formats) but the application focuses on receptive music therapy interventions (including song discussion, playlist construction, music-based self-management) or active music therapy interventions (songwriting) that can be delivered in a virtual format (minor).
- Congruent with other psychosocial intervention studies, it is not possible to mask participants to condition (minor).
- Cronbach's alpha values were not provided for the Spanish version of the instruments (minor).

Criterion 4: Investigator(s) and environment (Scientist Reviewers)

Strengths:

- The study team is comprised of leading experts in integrative medicine, oncology, CBT, MT, psycho-oncology, telehealth, statistics, and qualitative analysis. The group has considerable clinical and research expertise that are documented in the application (major).
- The PI has experience in PCORI research and has received funding from PCORI, National Institutes of Health (NIH), the Department of Defense, and the American Cancer Society (major).
- Other investigators have considerable experience in securing funding and conducting and publishing high impact

research. The multidisciplinary team is very strong and each member brings their expertise to the group. The governance structure is justified and the percent effort for all team members is appropriate (major).
- The settings, facilities, agreements, and resources are absolutely sufficient. There are a multitude of resources at the Memorial Sloan Kettering Cancer Center (ranked as one of the top two cancer centers in the US) and at Drexel University and Thomas Jefferson University. There is considerable support for this project from all who are involved as evidenced by multiple letters in the appendix (major).
- The research team has experience delivering services in virtual formats (minor).

Weaknesses:

- None - thank you.

Criterion 5: Patient-centeredness (All Reviewers)

Strengths:

- The application notes the many problems that cancer survivors experience including anxiety, depression, fatigue, insomnia, and quality of life. These are the primary (anxiety) and secondary (depression, fatigue, insomnia, and quality of life) outcomes of the proposed study and constitute critical gaps in the existing literature that are highly relevant to patients and other stakeholders. Additionally, the proposal includes a qualitative/interpretivist arm for both conditions that may provide additional patient-centric insights into virtual CBT and virtual MT (major).
- The application includes information about the benefits to patients and this is supported by the existing literature. As with all research, there is potential for harm but harm is unlikely given the nature of CBT and MT. The application has a plan should such harm occur (major).
- The application contains five letters of support from cancer survivors who are patient partners. There are also letters of support from the American Cancer Society and Society and Society for Integrative Oncology (major).
- While there are far more CBT practitioners than qualified music therapists, CBT and MT are currently offered to cancer survivors. Comparing MT to CBT – a treatment that has a large base of empirical support – has the potential to provide convincing evidence to the scholarly community and stakeholders and enable MT to be a treatment of choice for cancer survivors. In the literature, patients generally accept and favor MT (major).
- The study is available in both English and Spanish (major).
- The inclusive study will involve underserved and marginalized communities (goal is 30% of participants will identify as a minority) and can be delivered in English or Spanish to survivors of any type of cancer (major).

Weaknesses:

- Communities who are not as affluent may have difficult attaining a device to operate Zoom (minor).

Criterion 6: Patient and stakeholder engagement (All Reviewers)

Strengths:

- The application notes the degree to which the researchers have already actively engaged and involved patients and stakeholders as well as a plan for continued stakeholder involvement during the data collection period. The application describes how the study has involved patients, clinicians, hospital and health system representatives to ensure the study receives a wide range of input. The authors are obviously committed to patients and stakeholders through soliciting their feedback and involving them through the various stages of the project.

These engagements are ongoing and germane to the study (major).
- The proposed engagement plan is appropriate and tailored to the study (minor).
- Roles are clearly described in figure 2 with patient co-investigators being equal to the PI. The organizational structure and resources will ensure that the research team will be able to engage patients and stakeholders through all aspects of the study (major).
- Table 3 on page 14: Patient and stakeholder advisory board members. People have various types of cancer and roles and all will have input throughout the study via regular engagement meetings (major).
- The application has budgeted funds allocated for patient partners Co-investigators to attend local and national conferences to share results. This increases the number of people who will have access to the results (minor).
- The proposal included budgeted funds for publishing results in a peer reviewed journal. While the application did not explicitly state which journals the authors would submit the results to, it is implied the open access option will be used so that all people would be able to access results. The application does not explicitly state this but this is assumed. In addition, as this project would be federal funded, perhaps open access is assumed (minor).
- The design and protocols were informed and endorsed by patient participants. The researchers had a meeting on December 14, 2020 to solicit input from patients and other stakeholders (major).
- The advisory board will ensure language is accessible and appropriate for various interested people (minor).
- The application was informed by cancer survivors. The application noted a patient/engagement stakeholder meeting on December 14, 2020 wherein feedback was received about the research questions, inclusion criteria, measures, and protocol. Two patient partners are co-investigators (moderate).
- The study includes people from underserved and marginalized communities who speak English and Spanish. On page 21, Table 7 provides estimated racial/ethnic and gender enrollment demographic data (major).

Weaknesses:

- The application does not contain a letter of support from the American Music Therapy Association (www.musictherapy.org) or the Certification Board for Music Therapists (www.cbmt.org) (minor).
- The application does not include input from cancer survivor caregivers or family members. This is likely due to the population itself being cancer survivors who may not rely on their caregivers or family members as much as people who are receiving cancer treatments. Since the proposal contains a great deal of input from patient groups, it seems that this group will be able to take caregivers and family members perspectives into account. (minor).

Does the application have acceptable risks and/or adequate protections for human subjects?

Yes

There are plans to refer participants to Memorial Sloan Kettering Cancer Center psychiatry services if participants experience distress; plans for adverse event reporting.

Overall Comments:

Due to ongoing improvements in oncology treatment, more people are able to survive cancer. However, cancer survivors still encounter significant distress including anxiety, depression, fatigue, insomnia, and quality of life. While CBT can positively impact these factors, CBT has limitations including stigma, being too demanding, and requiring mental and emotional stamina. MT is a preferred and effective intervention for people with cancer but high quality MT research with cancer survivors is necessary. As such, MT has the potential to fill a consequential gap in the literature base for cancer survivors. Therefore, the purpose of this 2 arm randomized effectiveness study is to compare virtual CBT and virtual MT on measures of anxiety, depression, fatigue, insomnia, and quality of life in adult cancer survivors (“MELODY trial”).

The study includes follow up at 8 and 16 weeks to determine maintenance of potential treatment gains. Virtual delivery ensures participants will be able to receive services during COVID and augments accessibility. The study also contains a qualitative/interpretivist arm to better understand the lived experience of participants receiving CBT and MT. The design is strong, congruent with PCORI guidelines, and the multidisciplinary study team are all experts in their respective areas. There are adequate resources, facilities, and prospective participants. The inclusive study will involve underserved and marginalized communities (goal is 30% of participants will identify as a minority) and can be delivered in English or Spanish to survivors of any type of cancer. Patients and stakeholders have been involved in all aspects of the design of the study and will continue to be involved throughout all stages.

While the authors provided thorough and theoretically supported rationales for the treatments, a potential weakness of the application is the dose: Seven virtual sessions of CBT or MT may not be adequate to induce a measurable change at posttest and follow-up. The application did not provide a rationale or supporting literature for the dose. Overall, the study has strong potential to enable additional access to both virtual CBT and virtual MT as psychosocial treatments of choice for cancer survivors.

Reviewer 2:

Criterion 1: Potential for the study to fill critical gaps in evidence (Scientist Reviewers)

Strengths:

- The application defines a need for evidence to support the use of music therapy (MT) to support cancer survivors, as 1 in 3 suffer anxiety due to consequences of cancer treatment. This anxiety impacts daily functioning and quality of life. With COVID-19 exacerbating symptoms for many, there is an even greater need for treatment, and treatment alternatives that are effective and accessible virtually. Moderate Strength
- While MT and Cognitive Behavioral Therapy (CBT) have been shown to be delivered virtually on a wide scale, they have not been compared to one another for effectiveness virtually, or otherwise. If successful this study will fill a gap in evidence as well as support interventions to decrease burden. Moderate Strength
- Some patients do not wish to receive CBT, or for reasons cannot, and MT can play an important treatment role. Thus proving it is at least non-inferior to CBT, and while being delivered virtually, is important. Additionally by measuring which intervention is best at improving co-occurring symptoms, such as fatigue, adds strength. Moderate Strength
- With a multitude of remote treatment options entering the health care system due to COVID-19, there is a need for information on effective treatments to help patients choose one over another, as well as for providers to recommend them. It will also be important for payers to have evidence to support MT's use virtually. Moderate Strength

Weaknesses:

- None noted.

Criterion 2: Potential for the study findings to be adopted into clinical practice and improve delivery of care (All Reviewers)

Strengths:

- Demand has been seen from the American Psychological Association (APA), which has called for comparative

effective research (CER) of CBT and other psychotherapeutic interventions. Furthermore, the APA states that such interventions have been disproportionately targeted for women with breast cancer (p.3), and more interventions need to be explored to target patients with other types of cancer. The National Academy of Medicine (NAM) recognizes psychosocial services as, "essential components of high-quality cancer care." (p.2) Moderate Strength

- Support for this study to compare virtual treatment with MT or CBT is witnessed in numerous letters from providers who also express that the virtual delivery of such treatments is, "Critical during and beyond the COVID-19 pandemic." (letters p.7) One practicing oncologist expressed, "major need for non-pharmacological treatment options such as MT and CBT" and that the research team's study is, "crucial to improve psychological well being and quality of life (QOL) for cancer survivors." (Letters p.10) Moderate Strength
- Patients have expressed the importance of this study in letters of support. One breast cancer survivor stated how well both MT and CBT worked for her and that finding ways to reach patients in underserved populations with integrative therapy through this study is a must, and that the dissemination of the results will significantly impact how survivors approach treatment for anxiety. (letter p.13).
- Patients report that CBT requires a lot of mental and emotional energy and may be demeaning and taxing for survivors. MT may provide a more easy-going, personally tailored treatment that allows for less patient drop out. Major Strength for end use.
- Results should be easily replicated and implemented across a vast amount of health systems. MT is already a proven and recognized intervention, and if found to be as effective as CBT when delivered virtually, it should be no more difficult to adopt and implement virtually. Furthermore, many health systems already have many social and psychosocial interventions running virtually, so adding MT should be simple. Major Strength
- Dissemination plans beyond traditional means have been well thought out and planned. Stakeholder partners will provide key information on how to get an easy to remember message out and have already drafted a multi-channel dissemination plan that will include social media, internet blogs, support groups, brochures, newsletters and patient websites to provide study results and information for patients. The American Music Therapy Association will also play a key role in disseminating results. Major Strength

Weaknesses:

None noted

Criterion 3: Scientific merit (research design, analysis, and outcomes) (Scientist Reviewers)

Strengths:

- A randomized control trial (RCT) was chosen for validity of effectiveness, controlling large variables and measuring confounders between exposure and the primary outcome. Moderate Strength
- The researchers state they used the PICOTS (population, interventions, comparator, outcomes, time, setting) framework to guide the study. Minor Strength
- The study will test the non-inferior of MT to CBT for anxiety while also testing which treatment is superior in addressing other symptoms that occur with anxiety, such as fatigue. Major Strength
- Sub groups by individual characteristics such as age, sex, race and education will be used to explore the differences of these attributes on decision making. Major Strength
- Patient reported outcomes will be collected either by Research Electronic Data Capture (REDCap) online, or over the phone by choice of participant. All assessments and study materials will be given in English and Spanish. Major Strength
- Scales used to collect patient reported outcomes seem appropriate. They include the hospital anxiety and depression scale (HADS), the Brief Fatigue Inventory (BFI), Patient Reported Outcomes Measurement Information System (PROMIS) and Insomnia Severity Index (ISI). The ISI and the BFI have proven success and high validity when used amongst cancer patients. Major Strength

Weaknesses:

- Incentive for participants is very low. Participants will receive nothing to participate in the actual intervention for 7 weeks, and only after an assessment at 8 and 26 weeks, as well as a 45 minute phone interview at week 8, they will receive a total of $100. The assessments will consist of 5 scales with are estimated to take a minimum of 30 minutes each time. Participants taking medication for anxiety will also need to complete medication diaries at 0, 8 and 26 weeks. This may lead to difficult enrollment and/or drop out. Minor Weakness
- It is unclear how the researchers will account for medication use. The application only says they will track medication use by, "asking patients to complete weekly medication diaries at 0, 8, and 26 weeks" (p. 10). It is also unclear how a weekly diary is to only be collected during 3 weeks of a 26 week study. Minor Weakness

Criterion 4: Investigator(s) and environment (Scientist Reviewers)

Strengths:

- Very impressive, well qualified team. Dr. Mao, Lead principal investigator (PI), is an integrative medicine and oncology provider with a focus on integrative complementary therapies, and has led large trials for mental health and focus on cancer, including past PCORI awards. Dr. Bradt is a music therapist with PHD in health studies who has been PI on NIH studies on mental health and pain, and is the chief editor for a MT journal. Other team members include a director creative arts provider who is physician of integrative medicine, a bio statistic with previous PCORI experience, and patient partners with lived experience with cancer survival, MT, research and advocacy. All other CO-I's have vast experience with integrative therapies, cancer and research. Many of the team members have previously worked together. Major Strength
- Memorial Sloan Kettering and its multiple sites has provided detailed support, and proposed plans for support seem very appropriate. Major Strength
- All partners and team members have pledged major enthusiasm and support for this project in their letters of support, and roles are clearly delineated. Major Strength

Weaknesses:

- 2 patient partners on the team, Macleod and Walker, will receive substantially different compensation for what appears to be equal qualification, expertise and effort**.** Minor Weakness
- There is no one on the team representing Miami Cancer Center. Minor Weakness

Criterion 5: Patient-centeredness (All Reviewers)

Strengths:

- If successful, the study will provide evidence that MT is no less effective in treating anxiety virtually than CBT, but will also focus on which treatment is better at addressing other symptoms that occur with anxiety such as fatigue. Very patient centered in addressing co-occurring symptoms. Major Strength
- Both CBT and MT are available today and have been made available virtually due to the current pandemic. While both are proven effective as non-pharmacological treatments for anxiety upon cancer patients, CBT is more widely recognized as the first line treatment for anxiety. More evidence is needed to support that MT is just as effective for those who do not prefer CBT or are unable to keep up with the demands of CBT due to illness. It is very clear that patients are concerned about fatigue due to treatment demands, furthering the patient centeredness of this proposal. Major Strength

- The proposed study plans to answer questions that are important to patients (such as "which intervention is more effective for anxiety with co-occurring symptoms such as fatigue, and given my personal situation, which treatment deliver virtually will be better for me"). By examining variables such as age, sex, race and education, the study will be very patient centered and allow for answering the second question with even more precision. Major Strength
- The study hopes to provide evidence to support the use of MT as a non-pharmacological treatment for anxiety, citing that one in six survivors report using more than 2 psychotropic medications that are associated with poor QOL, financial drain, and higher risk of side effects and interactions. Finding evidence to support an alternative to medications may reduce this negative effects, which is very patient centered. Major Strength
- Interventions and comparators have been chosen based on demonstrated effectiveness as well as patient endorsement. Patients who have recently participated in MT virtually have expressed that the program has reduced barriers to access, fostered social connections and helped deal with stress, while increasing energy. Measuring all of these pieces in the proposed study to formally prove this is very patient centered. Major Strength
- The decision to focus on anxiety symptoms, rather than anxiety disorder, for the proposed study was based on conversations with patient stakeholders as well as via guidelines from the American College of Surgeons Commission on Cancer that used such guidelines on symptom severity, rater than psychiatric diagnoses. (P.6) Very patient centered. Moderate Strength
- Interviews with participants after the intervention period will focus on patient centered aspects of treatment such as, acceptability, impact on anxiety and coping, digital experience and any unanticipated benefits and/or harms. Major Strength
- Patient partners expressed need for evidence to help cancer survivors chose treatment that is best suited to their personal experience with anxiety and other co-occurring symptoms. Major Strength

Weaknesses:

- None noted.

Criterion 6: Patient and stakeholder engagement (All Reviewers)

Strengths:

- Patients and stakeholders were engaged prior to this application to inform proposal, identify research questions and the aims of the study. Moderate Strength
- Two patient partners with lived experience with cancer survival and expertise in research and advocacy are are part of the research team. They will help with recruitment of, and dissemination to, underrepresented groups. They have already drafted a far reaching multi-channel dissemination plan via social media and across patient support groups. They will co-present results at local and national advocacy conferences. Major Strength
- An 8 person advisory board has been formed and will include 5 patients with lived experience across 4 different cancer groups, 2 clinical providers, and 1 community stakeholder from the American Cancer Society. They are outlined to provide input throughout the study on protocols and outcomes, recruitment, engagement and dissemination. Minor Strength

Weaknesses:

- Payers are an overlooked stakeholder group. Minor Weakness
- While Music Therapists are on the study team, there are none on the advisory board. Minor Weakness
- Decision making amongst partners is not described. Minor Weakness
- The advisory board will only meet 2x year. This is not sufficient enough time for engagement and input

throughout the study in all areas as outlined. This is also not consistent with the researchers statement in the application that, "hosting regular engagement meetings will ensure observance of the PCORI Principle….and allow all major decisions regarding study." (p.14) Minor Weakness

- Advisors will only be paid $50 a meeting. Additionally they will be required to take a 6 hour research training for which they will only be compensated $150.00. Minor Weakness

<u>Does the application have acceptable risks and/or adequate protections for human subjects?</u>

Yes

<u>Overall Comments:</u>

The proposed study will compare two interventions (CBT and MT, delivered on virtual platforms) for treating anxiety symptoms in cancer survivors,. While both interventions have proven effective, CBT is the recommended first line treatment, and the interventions have not been compared virtually or otherwise. If successful, the study hopes to prove that MT is no less effective then CBT when delivered virtually, and hypothesizes that MT will be more effective at decreasing fatigue. The researchers have chosen to study these interventions with cancer survivors specifically because they cite that this group of patients has the latest growing rate of anxiety and co-occurring symptoms such as fatigue.

Letters of support and meetings with survivors confirmed the need for alternative treatment to CBT for their anxiety as CBT can draw energy and increase fatigue. This proposal could fill a gap in evidence to support the use of MT virtually on a wider scale and increase adoption, implementation, and coverage. With many health systems delivering care virtually due to the current pandemic, it should be easy to replicate the results and implement into practice. With many choices for virtual treatments today, this study may also help patients and their providers, choose a program that is best for them, based on their personal preferences and needs, knowing that one choice is no less effective on anxiety symptoms.

The study team is very strong, with an experienced PI who is an integrative medicine provider and oncologist who focuses on complimentary therapies such as MT, acupuncture and massage. CO-I's consist of experienced researchers, music therapists, biostatisticians, and most have successful past PCORI and/or NIH funded research. Two very experienced patient partners round out the team and are a strong asset. Support is seen from all partners, as well as from multiple sites, providers and organizations. The proposal is very patient centered with its focus on symptoms and interventions that are important to patients.

The engagement section of the application however is very small and mostly outlines players and vague activities, which is what brings the score down from excellent to very good. The advisory board may be missing a payer representative, and they will only meet 2x year to inform all aspects of the study, and how decisions will be made has not been addressed. Additionally board members will only received $50 compensation a meeting, and will be expected to take a 6 hour research training for which they will receive $150. Pay is similarly low for study participants. If the engagement plan were strengthened and better described it would make for a stronger proposal for what seems to be a very promising study otherwise. If successful, the study would be generalizable to a broad range of patients experience anxiety symptoms, not just cancer survivors, and MT could join the rank of first line treatment recommendations and increase its use and reach.

<u>Reviewer 3:</u>

Criterion 1: Potential for the study to fill critical gaps in evidence (Scientist Reviewers)

Strengths:

- The proposal notes there will likely be over 22 million cancer survivors living in the United States by the end of this decade and that nearly one in three of these survivors suffer from anxiety symptoms indicating a substantial clinical burden. (Moderate)
- While a growing body of evidence indicates that both cognitive behavioral and music therapies (CBT and MT) are associated with greater reduction in anxiety among cancer survivors compared with usual care, the two therapies have not been directly compared. The American Psychological Association (APA) has called for "continued and further research on the comparative effectiveness" of CBT and other psychotherapeutic interventions, identifying a critical gap in current knowledge. (Moderate)
- The application identifies several gaps in knowledge that affect clinical decision making. First, not all individuals are able to complete a full CBT treatment course, a first-line treatment for anxiety, complicating treatment decisions. Second, people may be reluctant to pursue CBT due to the socio-cultural stigma surrounding psychotherapy in different communities. Finally, for people who do not respond or wish to pursue CBT, it remains unclear whether MT is an effective treatment option that is non-inferior to CBT, further complicating treatment decisions. (Moderate)
- Another key evidence gap is the lack of diverse representation in CBT trials. Most trial participants have been well-educated and white. (Major)
- The proposed randomized clinical trial would provide high quality evidence to compare the effectiveness of cognitive behavioral and music therapies to treat anxiety among cancer survivors. The proposal to enroll a racially/ethnically diverse, heterogenous population from urban, suburban, and rural settings to ensure that findings are applicable to diverse cancer survivors. (Major)

Weaknesses:

- None noted.

Criterion 2: Potential for the study findings to be adopted into clinical practice and improve delivery of care (All Reviewers)

Strengths:

- ·Stakeholder organizations the investigators are connected with, such as the American Cancer Society, may include the study findings in their discussions of treatment options for anxiety. (Moderate)
- The primary end-users of the comparative effectiveness results of this study will be the patients with anxiety who receive the cognitive behavioral or MT and clinicians who recommend therapies and the proposal includes extensive dissemination plans to engage both groups. (Moderate)
- The investigators cite studies that show that 20-25% of CBT participants fail to complete a full treatment course and that patient stakeholders commented that a full treatment course requires "significant mental and emotional stamina" that may be too demanding and taxing for some survivors indicating the need for alternative therapies for anxiety. The investigators also cite studies that support the use of MT to treat anxiety in cancer populations. Finally, several studies demonstrate that both cognitive behavioral and music therapies can be successfully delivered virtually. (Moderate)
- Demonstrating that virtual MT is as effective as virtual CBT to treat anxiety in cancer survivors could inform treatment decisions of both clinician and patient stakeholder by providing a treatment option that avoids the stamina and energy that CBT requires. Since the interventions of interest will be delivered virtually, it is very likely that others could reproduce the findings. (Moderate)
- The investigators propose to take advantage of the existing infrastructure at Memorial Sloan Kettering (MSK) and their patient and stakeholder partners to disseminate using social media, internet blogs, support groups, community outreach, informational brochures, newsletters, and patient websites to provide

other patients with treatment-related information based on study results. The investigators will also leverage their pre-existing relationships with stakeholder organizations such as the American Cancer Society to disseminate information to their members. These are solid plans that are likely to succeed. (Major)

Weaknesses:

- The proposal does not explicitly identify who will make decisions based on the comparative effectiveness results this study will produce but one would assume that clinician and patient stakeholders would use the results to guide treatment decisions. (Minor)
- Since the interventions require internet access, cancer survivors without such access will not be able to use the therapies. (Minor)

Criterion 3: Scientific merit (research design, analysis, and outcomes) (Scientist Reviewers)

Strengths:

- The proposal clearly describes the proposed randomized clinical trial design, the cognitive behavioral and music therapy interventions, the anxiety and other symptom outcomes and discusses relevant literature that support the investigators' research design. (Moderate)
- With a few minor exceptions, the overall Research Plan including the study design, subjects studied, outcomes investigated and statistical analysis plan closely adhere to the PCORI Methodology Standards. The focus of the statistical analysis on the time-by-intervention arm interaction is appropriate. (Moderate)
- The proposal well-justifies the choice of the randomized clinical trial study design as the most appropriate way to obtain a valid measure of effectiveness and control for confounding. (Major)
- The investigators appropriately propose to gather a diverse sample of cancer survivors from the MSK regional network in New York and New Jersey and from the Miami Cancer Institute in South Florida. The investigators aim to enroll >30% non-white participants and will rely on Miami Cancer Institute enhance Hispanic accrual. These are appropriate patient populations and the investigators have experience working with them. (Moderate)
- The investigators based their choice of outcomes on the scientific literature and input from patient partners on what aspects of their cancer symptom experience are important to them and will use measurement instruments that have been previously validated in both English and Spanish versions. (Moderate)
- The proposal clearly describes the cognitive behavioral and music therapy interventions and justifies the objective to compare them in a diverse sample. (Major)
- The inputs into the sample size calculations are appropriate so that the proposed sample size is likely appropriate as well. (Moderate)
- The study plan seems feasible and the investigators have pilot tested the data collection and management system. The investigators are experienced with trials of cancer survivors and calculated reasonable estimates of potential patient pools and recruitment rates. The project timelines and milestones are all realistic. (Moderate)
- The investigators appropriately base their sample size calculations on tests of the time-by-intervention arm interaction. (Minor)

Weaknesses:

- The investigators do not describe or cite the methods they used in their sample size calculations. The proposal also needs sample size calculations to describe the magnitudes of the heterogeneity of treatment effects their proposed sample will be able to detect with high power. (Minor)
- The plans to plot the outcome measure trajectories by randomization arm over time and summarize each

outcome measure at each assessment time by treatment arm do not seem to focus on the trajectories of individual subjects which is the objective of the statistical analysis. (Minor)

- The proposed linear mixed effects model analyses are not completely clear. For example, the investigators do not clearly describe exactly which time points they will include in the analyses nor how they intend to model the time-by-intervention arm interaction. Overall, the proposal focuses on statistical tests and does not mention plans to assess magnitudes of intervention group differences using quantities such as 95% confidence intervals. (Minor)

Criterion 4: Investigator(s) and environment (Scientist Reviewers)

Strengths:

- The research team consisting of the principal investigator (PI), Dr. Mao, the co-PIs, Drs. Bradt and Trevino, co-investigators Drs. Lopez and Panageas is well-qualified to carry out the proposed research and several of the investigators have worked together in the past. The research team includes complementary expertise in integrative medicine, cognitive behavioral and music therapies, statistics and telemedicine. (Major)
- The PI, Dr. Mao, is experienced in designing and executing clinical trials aimed at alleviating physical and psychological symptoms and improving quality of life in cancer patients. These trials were of a similar size, scope, and complexity as the proposed trial. (Moderate)
- The proposed levels of support for Drs. Mao, Bradt, Trevino and Lopez are appropriate and well-justified. (Moderate)
- The Physician-in-Chief at MSK, Dr. DeAngelis, wrote a letter of support for this project but did not offer any specific resources. (Minor)
- The research facilities and resources at Memorial Sloan Kettering Cancer Center (MSKCC) and the Miami Cancer Institute are excellent will support the successful completion of the proposed research. (Moderate)

Weaknesses:

- The proposed 1.2 calendar months effort for Dr. Panageas is not sufficient since she will be responsible for conducting complex statistical analyses as well as overseeing the work of Mr. Baser. (Minor)
- The proposed 3.6 calendar months effort for Dr. Liou is not well-justified since his duties duplicate the roles of Dr. Mao and Ms Seluzicki. (Minor)
- The Lead Music and Lead Cognitive Behavioral Therapists will play major roles but the application does not identify the people who will perform this work and their expertise cannot be evaluated. (Moderate)

Criterion 5: Patient-centeredness (All Reviewers)

Strengths:

- The investigators note that discussions with patient stakeholders revealed that anxiety symptoms represent an outcome that survivors notice and care about. (Moderate)
- The investigators note that when they engaged their patient stakeholders, those with prior CBT experience commented that a full treatment course requires “significant mental and emotional stamina” that may be too demanding and taxing for some survivors suggesting that patients are interested in alternative, effective treatments. Further discussions with patient stakeholders revealed that many of them used music to cope with difficult emotions during their cancer journeys, indicating interest in MT. (Minor)
- The video-based music and cognitive behavioral therapy protocols were developed based on prior research of

the study team and the extensive literatures on music and cognitive behavioral therapies for anxiety. The investigators suggest that there is an important need for effective mental health treatments that everyone can easily access such as the interventions to be studied in this project. (Minor)

Weaknesses:

- The proposal does not mention that these interventions are available to patients right now. (Minor)

Criterion 6: Patient and stakeholder engagement (All Reviewers)

Strengths:

- The project will appropriately include two patient co-Investigators, Ms. Macleod and Ms. Walker, as well as an advisory board consisting of patients representing diverse cancer experiences, as well as key clinical and community stakeholders. All patient and stakeholder partners provided letters of support describing their roles for the proposed project and these roles are appropriate. (Major)
- The investigators actively engaged patient and clinical stakeholders in the development of the project and will continue this engagement during the execution of the project. Patient and clinical stakeholders provided guidance in areas such as the subject inclusion and exclusion criteria, follow-up time and study outcomes. The project scientists and patient stakeholders also have joint publications. The investigators appropriately plan to hold bi-annual meetings with their patient/stakeholder partners to review study progress, develop or revise recruitment and engagement strategies, and plan for dissemination efforts. The bi-annual meetings seem frequent enough to allow the clinical and community stakeholders to provide input and perspective. (Moderate)
- The proposed Engagement Plan appropriately includes cancer survivor co-Investigators as well as an appropriately chosen advisory board consisting of patients representing diverse cancer experiences, as well as key clinical and community stakeholders. Of note, the PI and research team (including two patient partner co-Is) have extensive experience with patient and stakeholder engagement in past and ongoing research projects. (Moderate)
- The proposal clearly describes the roles of the patient co-Investigators and the advisory board. Ms. Macleod and Ms. Walker will contribute their patient perspectives and play key roles in patient engagement and outreach efforts. (Moderate)
- The proposal clearly describes the organizational structure of the study team and provides appropriate financial support to both patient co-Investigators throughout the project. (Moderate)

Weaknesses:

- None noted.

<u>Does the application have acceptable risks and/or adequate protections for human subjects?</u>

Yes

<u>Overall Comments:</u>

The objective to compare the effectiveness of virtual cognitive behavioral and music therapies to reduce anxiety among

cancer survivors using a randomized clinical trial is well-motivated by patient concerns and will provide high quality results. The research team is strong with complementary expertise in integrative medicine, cognitive behavioral and music therapies, statistics and telemedicine. The PI, Dr. Mao, is experienced in designing and executing clinical trials aimed at alleviating physical and psychological symptoms, as well as improving quality of life in cancer patients and has led previous PCORI studies. Patients provided useful input into the development of the project which will include two cancer survivor co-Investigators. The plans to disseminate the study results are solid and the comparisons of treatments that can be delivered virtually are timely with the move toward telemedicine, especially given the current pandemic.

On the downside, the roles of some of the co-Investigators are not well-justified due to duplication of effort with other investigators. In addition, the proposal fails to identify the Lead Music and Lead Cognitive Behavioral Therapists will play major roles in the delivery of the therapies. The project also does not devote sufficient resources to statistics. Finally, the proposal devotes insufficient detail concerning the sample size calculations and an inadequate description of the proposed linear mixed effects model analysis.

Reviewer 4:

Criterion 1: Potential for the study to fill critical gaps in evidence (Scientist Reviewers)

Strengths:

Weaknesses:

Criterion 2: Potential for the study findings to be adopted into clinical practice and improve delivery of care (All Reviewers)

Strengths:

- The decision makers who will be able to utilize the results of this research project to make health care choices will be cancer survivor patients who are experiencing anxiety, their families and caregivers and their treatment providers such as oncologists, counselors, psychologist and doctors. Major Strength
- The results of this study will also be utilized by the end-users at auxiliary professional organizations such as the American Music Therapy Association (MTA), the American Psychological Association (APA), American Society of Clinical Oncology (ASCO), the National Comprehensive Cancer Network (NCCN), American College of Surgeons Commission on Cancer, American Cancer Society, Memorial Sloan Kettering Cancer Center (MSKCC), Drexel College of Nursing, Sydney Kimmel Cancer Center at Thomas Jefferson University and various medical and patient advocacy groups. Major Strength
- Representative end-users such as these will be engaged partly in the Patient/Stakeholder Advisory Group who will participate in all phases of the research project and partially through the dissemination of results. Moderate Strength
- The Co-Investigators, Dr. Bradt and Dr. Trevino, are affiliated with many of these organizations and other cancer centers and plan to leverage these organizations to disseminate results to these end-users as well. Major Strength
- Patients, providers and professional organizations contributed to the selection of Musical Therapy (MT) and Cognitive Behavioral Therapy (CBT) for a comparative effectiveness study with virtual treatment delivery and have input throughout the formulation of the research question and methods. These end-users expressed concern about the adverse effect of polypharmacy in current practice. the effectiveness of CBT with the underserved and diverse population groups (previous highest success rate has been associated with

white, highly educated, males), and desired further research on comparing virtual CBT with other virtual methods for treatment of anxiety. Major Strength

- Stakeholder concerns with the increasing population of cancer survivors, the increasing use of virtual health care delivery throughout the medical community, and the lack of research on CBT for a diverse population contributed to the selection of these study treatment modalities and delivery methodology. Major Strength.
- Selection of a virtual treatment modality will facilitate easier usage by providers and patients and avoid close contact in healthcare in the context of the COVID pandemic. Major Strength
- This research plan includes a dissemination plan focused on many layers of end-users and many modalities of delivery. The patient partners have drafted a multi-channel dissemination plan utilizing various forms of social media to target other patients. The Integrative Medicine Service at MSKCC has an award winning website, "About Herbs" and monthly e-newsletter that targets patients, clinicians and the public throughout the country and will be utilized to disseminate study updates and findings. Study findings will be distributed to study participants at the study's conclusion and they will be invited to the annual cancer survivor conference held each year. Major Strength.
- The protocols and implementation plan for the project will be formalized and available for replication and the outcome measurement tools are already validated and in practice for ease to replicate. Moderate Strength.

Weaknesses:

- The Music Therapy intervention is explained as moving from initially developing a relationship of trust and then moving from a passive role involving music (listening) to a more active role such as developing a play list, singing and writing songs. Singing and writing songs require an integrated cognitive activity level and it would be difficult to engage all personalities in this method. One of the aims of this study is to include underrepresented populations and diverse groups of patients and it may be difficult to engage all randomly selected patients to engage in this level of social and mental activity. Minor Weakness.
- A barrier to reproduction would be the challenge of standardizing CBT for comparison purposes. While the time frame and schedule has been standardized, the methodology is much harder to replicate for transference. Minor Weakness.

Criterion 3: Scientific merit (research design, analysis, and outcomes) (Scientist Reviewers)

Strengths:

Weaknesses:

Criterion 4: Investigator(s) and environment (Scientist Reviewers)

Strengths:

Weaknesses:

Criterion 5: Patient-centeredness (All Reviewers)

Strengths:

- Prior to and during the formulation of the proposal, patients expressed a desire to have at least some qualitative outcome measures to address the quality of life issues associated with anxiety and also a desire to focus on the symptomatology of anxiety as opposed to being evaluated for a clinical disorder of anxiety. Those concerns were incorporated into the research plan and will be partially expressed with semi-structured interviews instead

of quantative outcome measures exclusivly. Major Strength
- Patient and stakeholders also expressed the opinion that CBT might require a more significant effort, may be too demanding, contribute to social stigma and that members of the underrepresented population are more likely to drop out and would be interested in an alternative treatment. Major Strength
- Patients requested that patients currently taking medications be included in the study and this was incorporated into the final proposal. Major Strength
- Patients and stakeholders noted the lack of representation of underserved populations in previous studies and sought to close the evidence gap by advocating for recruitment of a representative sample of actual patients including different sexes, different ethnic and racial backgrounds, socio-economic conditions, and different types of cancer. Major Strength.
- The proposed interventions of MT and CBT have both been utilized in the field of behavioral health and both have been found to have positive outcomes. The comparison of these treatments has not been done using a virtual modality. The increased use of virtual treatment due to COVID, accessibility and cost of healthcare, has facilitated the need for more research using virtual modalities. This proposal stands to leverage the increase in virtual treatment and to test its' effectiveness with two existing therapies. Major Strength.
- Patients and stakeholders contributing to this project expressed the barriers to some patients in engaging and adhering to CBT and welcome an alternative treatment modality. Moderate Strength.
- Involved patients expressed the desire that the follow-up be more of a long term time frame and that was also incorporated into the proposal. Minor Strength.

Weaknesses:

- MT is not available in all cancer centers. Minor weakness.

Criterion 6: Patient and stakeholder engagement (All Reviewers)

Strengths:

- This proposal's research team will build upon an existing patient, stakeholder and community organizational relationship with MSKCC. The research plan includes a Patient/Stakeholder Advisory Board which met on December 14, 2020 to initiate plans and procedures for this proposal, including patient centered research questions, appropriate inclusion and exclusion policies, research protocol, and appropriate outcome and measurement tools. Patients representing different cancer experiences were included in the Board's makeup. This Board will communicate regularly but meet in person twice a year with the research team and the patient Co-Investigators (co-Is). Their responsibilities are indicated to include providing ongoing feedback throughout the project including the research protocol, ongoing recruitment and engagement strategies and dissemination efforts. Major strength
- The application indicates that the patient stakeholder advisory board will monitor adherence to the PCORI Principles of Partnerships guidelines. Major strength.
- The organizational structure is adequate to support activities and include patients throughout the process of implementation. There are two patient co-investigators named that have had experience in previous research. Moderate strength.

Weaknesses:

- Even though the patients have been designated as co-Is, the description of their responsibilities focus' on recruitment and dissemination. There is no mention of how they will interface with the research team on implementation factors. Minor weakness.
- There isn't a description of how the Advisory Board will initiate ongoing communication and oversight since they

meet with the research team and patient co-Is twice a year. There's no mention of how ongoing communication will take place. Minor weakness.
- There's no mention of auxiliary stakeholders such as payers, purchasers, psychiatrists, nurses, mental health counselors, and caregivers, being included on the Advisory Board or for input, . Minor weakness

Does the application have acceptable risks and/or adequate protections for human subjects?

Overall Comments:

Due to increased success in cancer treatment the numbers of cancer survivors have increased greatly and are projected to continue to increase at great rates. The physical and psychological burden of a cancer diagnosis is great initially, but those who survive and their families are typically left with a lot of anxiety inherent in the supposed random nature of cancer, factors involving capacity, fatigue, financial burden, and perhaps new limitations. There has not been established any certain method recognizing or a certain modality of treating this anxiety.

Patients and stakeholders indicate that the usual first line of mental health treatment, cognitive therapy and psychopharmacology, isn't always effective or desired. In conjunction with this project they called for an alternative approach, a more diverse research population and attention to desired qualitative outcomes such as quality of life and common symptoms such as fatigue. This study addresses and incorporates investigating these requests.
In general virtual treatments are increasing at a great rate and in many cases are highly desirable due to COVID, procedures cost, accessibility and ease. This proposal leverages this trend and tests its approach between two existing treatments and their virtual delivery. It stands to advance several different concepts of treatment.

There is some weakness in the proposal regarding the availability and standardized use of MT, the ability to replicate, and lack of some specific language regarding methodology for incorporating patient and stakeholder involvement in implementation with a seeming emphasis on recruitment and dissemination with their roles.

This proposal stands to benefit many people who have already suffered through a cancer diagnosis and are so deserving of help with the prolonged effects. Testing 2 potential treatments and advancing the field of virtual medicine make this a strong proposal.

Reviewer 5:

Criterion 1: Potential for the study to fill critical gaps in evidence (Scientist Reviewers)

Strengths:

- Major: The application indicates that anxiety is one of the most common mental health issues facing cancer survivors. About 30% of cancer survivors experience significant anxiety symptoms, which impair functioning and are associated with poor treatment adherence and worse quality of life.
- Moderate: The application indicates that no study has compared music therapy (MT) to cognitive behavioral therapy (CBT) for anxiety symptoms in cancer survivors.
- Minor: American Psychological Association (APA) and other organizations have called for more comparative effectiveness research of CBT and other psychotherapeutic interventions. This is only a minor strength because

these guidelines do not specifically identify the comparison of CBT vs. MT.

- Major: The recruitment of a racial and ethnically diverse sample could address the research gap regarding the effectiveness of CBT and other treatments in minority groups.

Weaknesses:

- None noted.

Criterion 2: Potential for the study findings to be adopted into clinical practice and improve delivery of care (All Reviewers)

Strengths:

- Major: The application notes the surge in telehealth, especially during COVID-19. The proposed virtual delivery of interventions is especially timely, potentially more accessible, scalable, and likely to improve care delivery.
- Major: The findings are likely to be reproducible given the rigorous methods. The findings are likely to be generalizable given the recruitment of a more diverse sample.
- Major: The inclusion of Spanish increases the reach of these interventions to a growing minority group.
- Minor: The dissemination plan includes generic themes such as multi-channel dissemination, social media (e.g., Instagram, Facebook, Twitter, and YouTube), internet blogs, support groups, community outreach, informational brochures, newsletters, and patient websites. Among these, the application explicitly indicates the website "About Herbs".

Weaknesses:

Weaknesses:

- Minor: The application does not provide information that supports the demand for this kind of study from end-users and does not identify who will use the study's findings. It seems assumed that clinicians and other stakeholders will use the study's findings.

Criterion 3: Scientific merit (research design, analysis, and outcomes) (Scientist Reviewers)

Strengths:

- Major: The application provides a clear conceptual framework for the proposed study supported by relevant background literature and the investigators' past experiences with CBT and MT.
- Major: The proposed randomized controlled study is well justified and adheres to the PCORI Methodology Standards. The trial will follow the Consolidated Standards of Reporting Trials (CONSORT) guidelines for non-pharmacological interventions. The blinding and the matching of time for MT and CBT are examples of rigorous methods.
- Major: The application identifies an appropriate study population (cancer survivors experiencing anxiety symptoms) and clearly describes the inclusion and exclusion criteria.
- Major: The primary (anxiety subscale from the Hospital Anxiety and Depression Scale [HADS]) and secondary outcomes are well justified and assessed with valid and reliable measures.
- Major: The application provides clear and convincing evidence that the two comparators are justified. For

example, they report on the effectiveness of CBT and MT. Professional cancer societies and clinical guidelines endorse the treatments.

- Major: The sample size and power analysis are based on estimates from past studies. The analytic framework (linear mixed-effects models) and approaches to missing data are clearly described and justified.
- Major: The study seems feasible, and it has realistic assumptions about subject enrollment, timeline, and attrition (15%). Evidence from a previously funded PCORI trial (recruitment of 27.5% of Black participants; 10% withdrew from CBT; <9% missing data) further supports the proposed plan's feasibility.

Weaknesses:

- Minor: The randomization should be stratified by sites to reduce risk of bias.
- Minor: The application does not describe the strategies used to recruit a diverse sample (besides recruiting from large and diverse metropolitan areas in NY and Miami).
- Minor: The exclusion of patients with cancer is not justified. While the inclusion of individuals with ongoing treatment would complicate the trial, these patients are likely to experience high anxiety and would benefit from treatments.

Criterion 4: Investigator(s) and environment (Scientist Reviewers)

Strengths:

- Major: The investigators have conducted a similar PCORI-funded trial for cancer survivors (comparing CBT to acupuncture). They have also published on MT. They have the required research, statistical, and clinical expertise.
- Moderate: The level of effort seems adequate (e.g., 20% for principal investigator (PI)). Figure 2 describes the organizational structure of the study team. The role and responsibility of each team member are clearly defined, complementary, and integrated.
- Moderate: The application indicates access to the planned study population, institutional resources and support, and collaborative agreements.

Weaknesses:

- None noted.

Criterion 5: Patient-centeredness (All Reviewers)

Strengths:

- Major: The application indicates that reducing anxiety, even if not necessarily an anxiety disorder, is important to patients.
- Moderate: The virtual assessment and delivery of treatments can address barriers to access to care. The application also noted the stigma associated with psychotherapy, which the virtual delivery could reduce. MT is likely to be less stigmatized than CBT.
- Moderate: CBT is a first-line treatment for anxiety and one of the most common psychotherapeutic interventions. The application notes that according to public-facing websites, most Comprehensive Cancer Centers offer some MT services.

Weaknesses:

- Minor: The application does not provide clear information that comparing MT to CBT is important to patients.

Criterion 6: Patient and stakeholder engagement (All Reviewers)

Strengths:

- Moderate: Two patient partners are included as co-investigators (co-Is) and five are in the patient/stakeholder advisory board. A patient/stakeholder advisory board is tasked with providing input on all aspects of the study and meets biannually with the research team.
- Minor: The application indicates that patient partners will receive appropriate compensation.

Weaknesses:

- Moderate: The engagement plan provides little information on the level of engagement from patients. Even the date (December 14, 2020) raises questions on the actual contributions of patients. Given the admirable intention to recruit from minority populations, the application provides little information on how the team will engage these groups. Also missing is information on whether the patient/stakeholder advisory board includes members from underserved communities.
- Moderate: The patient/stakeholder advisory board does not include caregivers or family members who play a vital role in cancer survivors' lives. There is limited engagement with mental health advocacy organizations.
- Minor: The application does provide limited information on the resources and roles of study partners. The engagement plan does not seem tailored to the study.

Does the application have acceptable risks and/or adequate protections for human subjects?

Yes

Overall Comments:

The proposed randomized clinical trial (RCT) tests whether music therapy (MT) is not inferior to cognitive behavioral therapy (CBT) for reducing anxiety among 300 cancer survivors. Professional cancer societies recommend the two treatments, but the application indicates that no comparative effectiveness research (CER) has compared these two interventions, particularly when delivered remotely. The trial will also test whether MT is superior to CBT at reducing fatigue. The application will explore additional aims, such as investigating whether baseline characteristics moderate treatment effects and conduct semi-structured interviews for a qualitative study.

The patient and stakeholder engagement seems weak, with little or no involvement of caregivers/family members, mental health advocacy groups, and other stakeholders besides a few patients.

Despite some minor and fixable weaknesses, the application is excellent, it is scientifically rigorous, and it has several notable strengths. The two well-matched treatments consist of seven 60-minute sessions delivered remotely. Even without a pandemic, such remote interventions can increase access and reduce treatment barriers for those who need or prefer therapy online. The planned inclusion of minority groups (>30%) and the Spanish-language version will help reach a broader population and provide more generalizable findings.

The proposed sample size, power calculations, recruitment, and attrition are based on past research experience (including a previous PCORI RCT) or published evidence. Previous work also indicates that the study is feasible, and the team can complete this work. Overall, the proposed CER will provide information on psychotherapeutic interventions that can improve cancer survivors' quality of life.

This is the end of the Summary Statement
